# Effect of trabecular bone loss on cortical strain rate during impact in an in vitro model of avian femur

**DOI:** 10.1186/1475-925X-5-45

**Published:** 2006-07-19

**Authors:** Tal Reich, Amit Gefen

**Affiliations:** 1 Department of Biomedical Engineering, Faculty of Engineering, Tel Aviv University, Tel Aviv 69978, Israel

## Abstract

**Background:**

Osteoporotic hip fractures occur due to loss of cortical and trabecular bone mass and consequent degradation in whole bone strength. The direct cause of most fractures is a fall, and hence, characterizing the mechanical behavior of a whole osteopenic bone under impact is important. However, very little is known about the mechanical interactions between cortical and trabecular bone during impact, and it is specifically unclear to what extent epiphyseal trabecular bone contributes to impact resistance of whole bones. We hypothesized that trabecular bone serves as a structural support to the cortex during impact, and hence, loss of a critical mass of trabecular bone reduces internal constraining of the cortex, and, thereby, decreases the impact tolerance of the whole bone.

**Methods:**

To test this hypothesis, we conducted cortical strain rate measurements in adult chicken's proximal femora subjected to a Charpy impact test, after removing different trabecular bone core masses to simulate different osteopenic severities.

**Results:**

We found that removal of core trabecular bone decreased by ~10-fold the cortical strain rate at the side opposite to impact (*p *< 0.01), i.e. from 359,815 ± 1799 μm/m per second (mean ± standard error) for an intact (control) specimen down to 35,997 ± 180 μm/m per second where 67% of the total trabecular bone mass (~0.7 grams in adult chicken) were removed. After normalizing the strain rate by the initial weight of bone specimens, a sigmoid relation emerged between normalized strain rate and removed mass of trabecular bone, showing very little effect on the cortex strain rate if below 10% of the trabecular mass is removed, but most of the effect was already apparent for less than 30% trabecular bone loss. An analytical model of the experiments supported this behavior.

**Conclusion:**

We conclude that in our *in vitro *avian model, loss of over 10% of core trabecular bone substantially altered the deformation response of whole bone to impact, which supports the above hypothesis and indicates that integrity of trabecular bone is critical for resisting impact loads.

## Introduction

Hip fractures owing to osteoporosis are common among elderly, and involve pain, loss of independence, reduced life expectancy, and substantial healthcare costs [1-6]. In biomechanical terms, the risk for an osteoporotic hip fracture is determined by the combination of factors inherent to the bone quality (e.g. cortical thickness, mineral contents, trabecular bone density and microarchitecture), as well as by the mechanical loading conditions caused due to a fall. Although the macroscopic size and shape of the femur are not changed substantially by osteoporosis, the degradation in quality of an osteopenic bone is expressed in both the cortical and trabecular components. For example, osteopenic bone loss in the femoral head can reach 50% of the normal trabecular bone mass, and 35% of the normal cortical bone mass (through reduction in cortical thickness) [7,8]. However, the microarchitectural loss of mass, thickness and structural integrity in trabecular bone have been particularly recognized as major risk factors for osteoporotic fractures [9]. Unfortunately, it is unclear how the osteopenic trabecular bone interacts mechanically with the bone cortex under impact loading. In other words, it is unclear to what extent does trabecular bone contribute to impact resistance of whole bones. Accordingly, this study follows the recent work of Passi and Gefen [10] in an attempt to better understand the role of trabecular bone in resisting impact loads applied to whole bones.

In our previous paper [10], we showed a statistically significant correlation between loss of trabecular bone mass and a decreasing energy that is required to fracture a chicken femur model. In the present study, we extended that previous work and compared cortical strain rates during impact in chicken femora with increasing levels of trabecular bone loss, by instrumenting the bone test specimens with strain gauges. Considering that trabecular bone may act as a scaffold to support and constrain the bone cortex from the inside during impact, we hypothesize that the failure pattern of an osteoporotic bone differs from that of a normal bone, and that this manifests in cortical strain rates during impact, which, in turn, also depend on the mass and integrity of core trabecular bone.

## Methods

Chicken femora were obtained fresh from a local slaughterhouse. All femora were harvested from battery-raised female chickens, older than 10 months of age, with completely ossified bones. Each femur was carefully cleaned from enveloping muscle tissue, external layer of cartilages and tendons, while taking care not to damage the bone cortex. Femora were then carefully sorted to exclude bones that were damaged in the slaughterhouse or during cleaning. Clean femora were sorted to form a group of specimens with similar weight and size. We thus composed a 21-specimen group with weight of 9.3 ± 2.1 g (mean ± standard deviation) and length of 7.7 ± 0.5 cm. Bones were then cut transversally at the diaphysis to fit the holding jigs of the pendulum-based impact testing system (Dynatup POE 2000, Instron Co.). Diaphyseal marrow was carefully cleaned through the open end of the medullar canal, using water and air pressure, with special care not to damage epiphyseal trabecular bone. Specimen weight was measured again after marrow removal, and in 18 specimens epiphyseal trabecular bone was subsequently removed through the open end of the medullar canal using a specially-designed hard tool with protruding tooth, as described in our previous work (Fig. 2 of Ref. [10]). Final weighing quantified the weight of trabecular bone that was artificially removed (i.e. net trabecular bone tissue weight, not including marrow). Range of removed trabecular bone weight was 0.08 to 0.47 g (resolution of the precision digital scale: 0.01 g). We took care to extract trabecular bone from the same site across different specimens, to avoid interference due to inter-site mechanical property differences in trabecular bone [11]. Hence, trabecular bone was mostly removed from the central epiphysis [10]. The other 3 bone specimens, from which trabecular content was not removed, served as controls. The test-ready specimens were frozen to -20°C and kept frozen for less than 14 days until the day of experiments. The literature shows that whole bone, cortical bone and trabecular bone mechanical properties are not affected by -20°C freezing for up to 100 days [12,13]. On the day of experiments bones were thawed to room temperature for two hours, during which they were kept moist.

**Figure 1:**
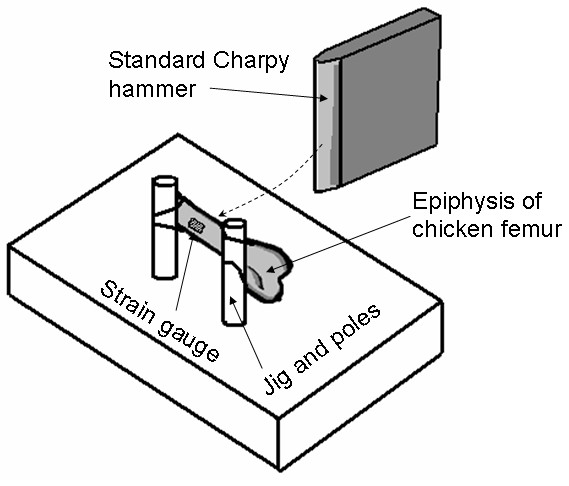
The experimental setup: Low Impact Pendulum Testing Machine with Charpy hammer that strikes the epiphysis. The strain gauge is attached on the anterior aspect of the proximal femur.

**Figure 2:**
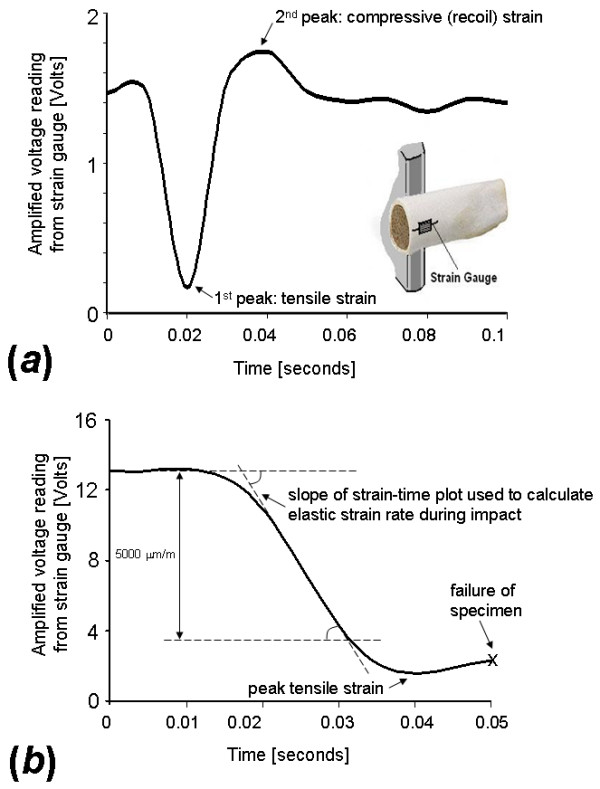
Strain signals measured on the cortex of a proximal epiphyseal chicken femur at the opposite side to the impact (as shown in the right bottom frame of plot (a)) using the experimental apparatus described in Fig. 1. This setup delivers bending-related tensile strains to the bone cortex (and coupled strain gauge) at the instant of impact. Hence, a in strain-time plot (a) for a control (unaltered) bone specimen which was subjected to impact energy of 0.3 joule, and did not break, a first peak shows tensile strains which are then counteracted by some elastic recoil response from the bone (second peak). This bone specimen was not included in further data analyses, and its data are shown here to illustrate the loading state in the bone during trials, and in particular, the decay of dynamic strains post-impact and the elastic recoil response of the bone. The strain-time plot (b) shows data for a specimen from which 0.12 g of trabecular bone were removed (~17% of the trabecular mass). The point of specimen failure and the part of the strain-time curve used to determine the strain rate are depicted. Scale of strains: 1 Volt = 500 μm/m.

Osteoporotic fractures are most often seen in the femoral neck. However, the size of the chicken femur (see Fig. 1 in [10]) did not allow good-quality attachment of a strain gauge to the femoral neck, and the small volume of trabecular bone in the chicken's femoral neck did not allow accurate control of the relative weight of removed trabecular tissue. Accordingly, strain gauges were attached at the center of the anterior lower part of the epiphysis, below the femoral head and the greater trochanter (Fig. 1). Impact loads were delivered by a Charpy hammer connected to a pendulum (Dynatup POE 2000, Instron Co.), to the posterior side of the bone. Special attention was given to positioning the gauge along the line of expected fracture, and in front of the center of the hammer of the impact testing system (Fig. 1).

In order to ensure that the bonding between the strain gauge and bone will be strong enough to endure an impact load, we used two glue layers, rather than a one glue layer which is commonly used in bone strain studies [14]. First, we located the site for strain gauge attachment (Fig. 1) and at that site, we gently cleaned a bone region at approximately the size of the strain gauge from residual periosteum. We then enveloped the bone with moist cloth excluding the adhesion site, rinsed this site with ethanol, and dried it locally with hot air for 20 seconds, while taking care that the rest of the bone is kept moist. We then delicately smeared a thin layer of epoxy resin at the adhesion site (N24, by Henkel Spain Co., designed for underwater hardening), which was hardened and gently planed by a 600 grit sand paper, until bone was visible again. Second, a uniaxial strain gauge (EA-13-240LZ-120, by Micro-Measurement Co., Vishay Measurements Group, Rayleigh, NC, USA) was attached to the bone longitudinally with cyanoacrylate glue (Superglue 5 by Henkel France Co.) over the processed epoxy resin layer. We wired each gauge before attaching it to the bone in order to prevent thermal bone damage by the solder.

This method of strain gauge attachment was first verified using standard wood specimens with circular cross-section (diameter 8 mm). We measured elastic strain rates in these wooden specimens during impact tests using the same impact conditions (hammer release angle, contact speed and impact energy) applied to bone, and, before each experimental session, verified that strain rate results are reproducible and repeatable (within a ± 10% range) across specimens in three consecutive trials.

The impact force was produced by a pendulum of an impact testing machine (Dynatup POE 2000, Instron Co.). This machine releases a standard Charpy test hammer to hit a bone specimen which is mounted onto two round poles of stainless steel (with elastic rubbers), so that the specimen is subjected to a 3-point bending impact loading mode (Fig. 1). The hammer was consistently released from an angle of 60° with respect to a normal to the test bench, which produces a contact speed of 1.8 m/s and impact energy of 0.9 Joule. We selected these impact parameters so that the contact speed is in the range of human fall speeds reported by van den Kroonenberg et al. [15], and the energy is above the fracture energy of chicken femora, which was previously found to be 0.4 joule [10]. We ran additional preliminary tests to verify that these impact parameters always cause fracture of the test specimen.

Cortical strains measured during impact were amplified (PM106GB amplifier, National Instruments Co.) and sampled to a PC (using Labview 7, National Instruments Co.) at a frequency of 1000 Hz. The dynamic strains caused by the hammer-bone mechanical interaction typically decayed within ~0.1 s (Fig. 2), which is similar to durations of dynamic mechanical responses in simulated fall trials with cadaveric human femora [16]. The measurement apparatus was calibrated prior to each experimental session by shunt calibration over the range of expected elastic strains (0–5000 μm/m), and accuracy of ± 0.5% was verified. A constant voltage (2 V) was applied to the Wheatstone bridge connected to the strain gauge in order to minimize thermal drift of electrical components, and all measurements were taken within 30 seconds from "power on" for that reason. An example of a sampled impact experiment where no fracture occurred is shown in Fig 2a. The hammer-bone contact event causing elastic tensile strains (first peak on strain-time plot) as well as some elastic recoil compressive strains in the anterior bone cortex after hammer rebounds (second peak on strain-time plot) are clearly shown.

The sampled strain-time data were processed to calculate the strain rate occurring during the elastic phase of tensile deformation, before fracture begins (Fig. 2b). The yield strain for avian bone, calculated in previous studies using the 0.2% offset method, was reported to be above 6,000 μm/m [17]. Accordingly, our calculation of elastic strains during the rise-time of tensile loading phase (Fig. 2) was limited to strains below 5000 μm/m. It was important to conduct our strain rate calculations below this elastic strain limit, since post-yield inelastic deformations may affect bonding of the gauge and thus potentially introduce measurement errors [14].

Statistical analysis included curve fitting to characterize the relation of cortical strain rates to trabecular bone mass, and *F *tests to determine whether elastic strain rates during impact were affected by the amount of trabecular bone removed. Prior to statistical testing, each strain rate result was normalized by the bone's initial weight to compensate for the inevitable, minor size differences between test specimens. Results were first plotted as normalized strain rate versus the weight of trabecular bone removed. Since this plot indicated that a sigmoid function will provide a better fit than a linear, or a negative exponential function (both visually and in terms of an *R*^2 ^correlation coefficient), we used the least-squares method to determine the parameters of that sigmoid function (interestingly, sigmoid-shape relations are common in life sciences [18]). An analytical biomechanical model of the experiments, described in the Appendix of this paper, also supported the selection of a sigmoid function. Evaluation of the goodness of fitting was based on the *R*^2 ^correlation coefficient, and the corresponding two-tailed probability value *p *was calculated. Results were considered statistically significant for *p *values below 0.05.

## Results

Successful impact trials were obtained with 3 control specimens and 8 altered specimens (from which trabecular bone was removed). Our criteria for inclusion of a measurement result in further data analysis were that (i) a continuous strain-time signal was recorded from the time of impact until specimen failure (as in Fig. 2b), and that (ii) there was no visible separation between the glue layer of the strain gauge and the bone cortex post-fracture. Accordingly, we excluded 10 specimens from which trabecular bone was removed (wiring of 5 strain gauges did not survive the impact and signals were incomplete, and in other 5 specimens failure of the bonding was visible, indicating that the bone cortex and strain gauge did not deform together).

**Figure 3:**
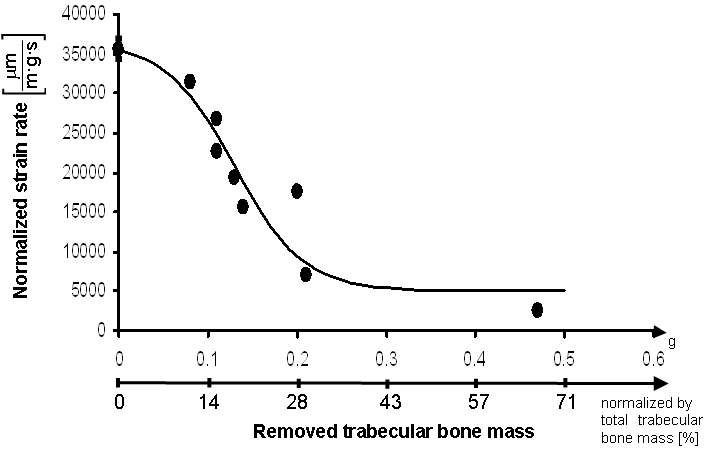
Strain rates normalized by the initial weight of bone specimens () versus the mass of trabecular bone removed (top horizontal axis: absolute mass in grams; bottom horizontal axis: normalized by the total mass of femoral trabecular bone in percent, *W*). Data point and error bar at *W *= 0 indicate the mean and standard error of measurements for 3 control specimens. Other data points indicate individual trials. A sigmoid function of the form  (where *a*,*b*,*c*,*k *are empirical constants) was fitted to the experimental data.



All bone fractures occurred at the site of impact, at the lower part of the epiphysis, below the femoral head and the greater trochanter. Cortical thickness of the fractured bones at this site was ~1 mm, and cortical bone occupied ~20% of the whole bone's cross-sectional area within the plane of the impact force (Fig. 1). The successful measurements indicated that elastic strain rates at the anterior cortex dropped significantly, by ~10-fold, with removal of core trabecular bone (*p *< 0.01). Specifically, strain rates dropped from 359,815 ± 1799 μm/m per second (mean ± standard error) for an intact (control) specimen down to 35,997 ± 180 μm/m per second where 67% of the total trabecular bone mass (~0.7 grams in adult chickens) were removed. We normalized strain rate data by the initial bone specimen weight, in order to account for the biological variability in initial weights of bone specimens (ratio of standard deviation over mean weight: ~23%). A plot of the strain rate normalized by the initial specimen weight, versus the trabecular mass removed normalized by the total trabecular mass in chicken femora (Fig. 3) indicated that the following sigmoid function provides adequate fit:

where  is the cortical strain rate normalized by specimen weight (in μm·m^-1^·g^-1^·s^-1^), *W *is the weight of removed trabecular bone normalized by the total weight of femoral trabecular bone (0.7 g in adult chickens), and *a*, *b*, *c*, *k *are the parameters of the sigmoid. Using the least-squares method, we found that the best fit (*R*^2 ^= 0.82, two-tailed probability value *p *< 0.01) is obtained for *a *= 18.1, *b *= 3.37, *c *= 4996 μm·m^-1^·g^-1^·s^-1 ^and *k *= 31478 μm·m^-1^·g^-1^·s^-1^. According to the empirical fitting in Eq. (1), the maximal strain rate that can occur on the cortex opposite to the site of impact, when *W *= 0, is _max _= *k*/(1+*e*^-*b*^)+*c *and the minimal strain rate, when *W *= 1, is _min _= *k*/(1+*e*^*a*-*b*^)+*c*. The decrease in the strain rate on the cortex opposite to the site of impact due to removal of the core trabecular bone, Δ, is therefore:



## Discussion

**Figure 4:**
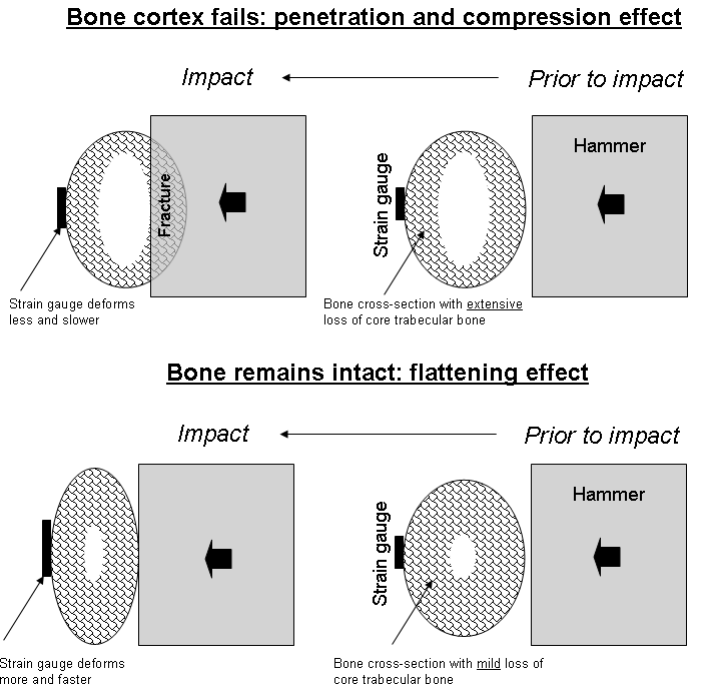
Schematic description of the internal constraining effect that trabecular bone has on cortical bone, and its contribution to resisting impact loads. If loss of core trabecular bone exceeds a critical mass, lack of energy transfer pathways causes impact stresses and strains to concentrate locally close to the site of impact, and the hammer penetrates the cortex, as shown in the top frame. If less than a critical mass of trabecular bone was lost, and sufficient number of trabecular pathways still exist to transfer strain energy away from the site of impact, the cortex deforms as a whole, as in the bottom frame, and, although it may crack, no catastrophic failure occurs. In this latter case, as more strain energy is transferred to the opposite side of the cortex, a strain gauge fixed to that bone aspect will deform more and faster with respect to the first scenario.

Overall, the results support our hypothesis that trabecular bone has an internal constraining effect on the cortex during impact, and thus, integrity of the trabecular contents is essential for resisting impact loads. The observation that loss of trabecular mass decreases cortical strain rates at the side opposite to impact suggest that less strain energy is transferred away from the hammer-bone contact site, since some of the trabecular pathways for strain energy transfer are disconnected. The outcome is likely to be more concentrated elevated strains and stresses at the hammer-bone contact site, which cause a collapse of the bone cortex inwards and a catastrophic failure of the whole bone. The internal constraining contribution of core trabecular bone to resisting impact loads in our experimental design is schematically depicted in Figure 4. If loss of core trabecular bone exceeds a critical mass, lack of strain energy transfer pathways causes impact stresses and strains to concentrate locally close to the site of impact, which generates local failures. This, in turn, allows the hammer to penetrate the cortex as shown in the top frame of Fig. 4. If less than a critical mass of trabecular bone was lost, and sufficient number of trabecular pathways still exist to transfer strain energy away from the site of impact, the cortex deforms as a whole, as in the bottom frame of Fig. 4, and, although it may crack, no catastrophic failure occurs. In this latter case, as more strain energy is transferred to the opposite side of the cortex, a strain gauge fixed to that bone aspect will deform more and faster with respect to the first scenario (see also the biomechanical model in the Appendix). We believe that this behavior is manifested in the -*W *relation in Fig. 3. Specifically, Fig. 3 shows very little effect on the cortex strain rate if less than 10% of the trabecular mass is removed, but most of the effect is already apparent for less than 30% trabecular bone loss. Accordingly, we conclude that in our avian impact model, loss of 30% of trabecular bone mass disconnected a critical number of load-transfer trabecular pathways. We also conclude, based on the sigmoid -*W *behavior in Fig. 3, that further loss (above 30%) of trabecular bone will have very little, if any, additional deteriorating effect on the deformation behavior of the whole bone under impact. This is expected since a minimal, critical number of strain energy transfer pathways is needed for spreading impact energy, and when the actual number of pathways drops below the critical threshold, a crush failure (as in the top frame of Fig. 4) is very likely.

Under real-world conditions of a fall, soft tissues that envelop the bone contribute to attenuation of the impact stress waves (which are mostly related to sudden bending loads [16,19,20] as in our present experimental design). While appreciating that soft tissue stress attenuation may have a substantial effect on the potential for inward crushing of the cortex (as in the top frame of Fig. 4), we still expect the trabecular pathways to play an important role in transferring strain energy away from the impact loading site in a real-world fall. Not only is the flow of strain energy through the trabecular microarchitecture important for clearing away the impact stress waves. Macroscopically, when considering the bone cortex during a fall as a tube subjected to sudden bending, it is evident from fundamental solid mechanics that a tube filled by (trabecular) material is more rigid in bending than a hollow tube of the same diameter (i.e. severely osteopenic bone). Therefore, before failure occurs, the cortex of an osteopenic bone that is directly subjected to an impact bending force deforms (as a whole) more and faster, compared with a normal bone. Loss of core trabecular mass thus affects bone deformation behavior at different structural scales, and, overall, decreases its tolerance to slow, fast and impact loads.

In this study, like in our previous paper [10], we used chicken femora as test specimens. We previously described similarities between morphological and biomechanical properties of chicken and human femora [10], and characterized the distribution of cortical and trabecular bone in the chicken's femur (Fig. 1 in [10]). Briefly, the volume fraction of trabecular bone in the proximal femur is around 15% in both species, cortical bone density is ~1.8 g/cm^3 ^for chicken and 1.5–2 g/cm^3 ^for humans, trabecular bone density is 0.3 g/cm^3 ^for chicken and 0.2–0.6 g/cm^3 ^for humans, and elastic modulus of cortical bone, 17–20 GPa, ultimate tensile strength, 100–300 MPa, and tensile strain to failure, ~6,000–13,000 μm/m, also overlap [17,21-26]. An important difference between chicken and human cortical bone is in fatigue strength (chicken bone has 60% the fatigue strength of human) [27], however, this is not influential in a single impact study design.

Since bone is, as all biological tissues, viscoelastic, a deformation-rate-dependent mechanical behavior should be expected. Thus, it is particularly important, when studying the mechanical interactions between cortical and trabecular bone as related to osteoporotic fractures, to study the bone when it is subjected to impact loading, as opposed to quasi-static loading. For example, Courtney et al. [28] showed that fracture loads can increase by as much as 20% as a result of a deformation rate change between 2 and 10 cm/s, whereas fall measurements show impact speeds of up to 3 m/s [15]. However, direct measurements of cortical strain, which are generally common in experimental bone mechanics [14], are technically problematic in impact studies. Specifically, the focal, large strains and non-continuity of bone material in the fracture area make it rather difficult to define the instant of onset of fracture. Additionally, the complex nature of the bone's biological structures makes it difficult to extrapolate real strains in sites other than the actual measurement site on the tested bone. Accordingly, previous impact studies of bone used either the strains prior to failure [16] or the external loads during fracture (without measuring real strains) to characterize the mechanics of fracture [19,20]. In order to allow inter-specimen comparisons without needing to define an instant of fracture for each specimen, we measured the elastic strain rate on the bone cortex during impact (prior to failure, Fig. 2b), rather than the yield strain or failure strain. Indeed, we found that strain rate is a more repeatable measure than the yield strain or failure strain. Nevertheless, we still had a relatively high rate of exclusion for our specimens, which demonstrates the technical difficulties in strain measurements during impact, since even minimal damage to the bonding layer (tolerable in common quasi-static or slow-loading measurements) may cause serious interferences during impact due to the intensity of stress concentrations at the geometrical irregularities of the bonding defects [14].

The literature contains several experimental evidences for the effect of trabecular bone loss on whole bone's mechanical behavior, however, nearly all data are relevant to quasi-static or slow loading trials. Specifically, Martens et al. [29], Werner et al. [30] and Delaere et al. [31] tested human femora and demonstrated that removal of the central part of the trabecular tissue at the proximal femur reduces the strength of the whole femur by about 40–50%, at both high loading velocities [29] and low loading velocities [30,31]. In a later study, by Rogers et al. [32], pairs of pigeon humeral bones were tested in slow compression after removal of inner trabecular tissue from one humerus of each pair (by means of a 2.5 mm hole at the head of the humerus). For slowly applied loads, it was found that deflection of the humerus lacking the trabecular tissue increased by 23%, while the bending moment at its base (fixed in epoxy) decreased by 21% and its work to fracture also decreased by 47% [32]. More recently, Ito et al. [33] evaluated the mechanical contributions of the spongiosa and cortex to the whole rat vertebra, using a finite element analysis system linked to three-dimensional data from microcomputed tomography. Their work also showed that trabecular microstructure has a significant relationship with whole bone strength. Last, using the same experimental model used in the present study – chicken's femora under impact – we previously showed that loss of over 0.5 grams of trabecular bone (approximately 71% weight fraction) reduced the energy required to fracture the whole proximal femur in mediolateral impacts from approximately 0.37 joule in controls to approximately 0.20 joule after extraction of core trabecular tissue [10]. Taken together, these studies, conducted in different species and at different loading velocities, consistently support our present hypothesis and experimental findings: the tolerances to fracture, as well as the failure mechanism in a whole bone, are strongly affected by the mass and integrity of trabecular structures. Trabecular bone therefore serves as a supporting scaffold to the cortex, not only in static, or slow loading, but also under an impact load, as in traumatic injuries.

Bone imaging modalities for studies of osteoporosis are constantly improving in resolution, and include, in addition to the pure measurement of bone mineral density (BMD), characterization of the microarchitecture of trabecular and cortical bones (usually in the spine and proximal femur), by means of quantitative computed-tomography (QCT) scanning [34-36]. In particular, studies which combined BMD and QCT measurements with mechanical testing found that the density and structure of trabecular bone alone are not sufficient to explain femoral fractures [37], which again indicates that the interaction between cortical and trabecular bone is a critical issue for understanding the failure of the femur. Future *in vitro *studies should therefore extend the present findings to correlate mechanical degradation in trabecular and cortical bones with clinically-accepted measures of the severity of osteopenia, such as BMD or QCT scans. For example, impact studies with human femora, subjected to a trabecular bone damage pattern which can be quantified in terms of trabecular bone mass loss, trabecular BMD loss (through BMD scans) and connectivity loss (through QCT scans), may allow to determine the critical trabecular mass and number of intact load-transfer pathways that are needed for the whole bone to effectively resist impacts. The clinical value of such analyses is in providing explicit baselines for trabecular bone quality when evaluating BMD and QCT scans of osteopenic patients.

In closure, our present results, although initial, show a strong correlation between mass of epiphyseal trabecular bone and the deformation behavior of bone cortex under impact. Taking the present findings together with our previous analysis of energy to fracture depending on the trabecular bone integrity [10], it becomes evident that mass and connectivity of the trabecular bone must be considered in assessment of the risk for an individual to suffer an osteoporotic hip fracture.

## Appendix

The purpose of this appendix is to demonstrate, using an idealized, simple analytical model of the experiments, that strain rate at the bone cortex opposite to the site of impact decreases with a decrease in the trabecular bone content.

**Figure 5:**
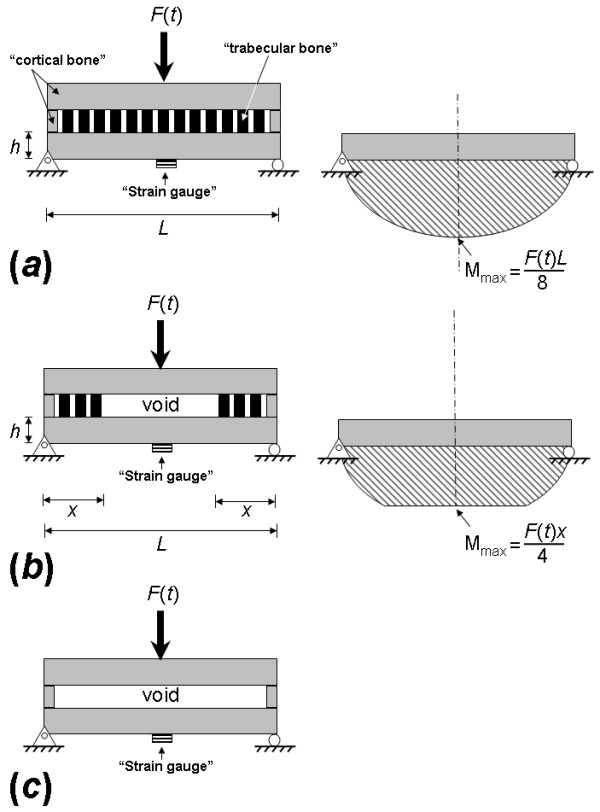
Idealized biomechanical model of the experiments: (a) "normal" bone, (b) mildly "osteoporotic" bone, (c) totally "osteoporotic" bone.



The model of a "normal" bone is described in Figure 5a. We assume that bone cortices are represented by elastic beams with rectangular cross-sections and lengths *L*. Hence, one elastic beam represents the bone cortex which directly faces the impact force *F*(*t*), and the other elastic beam represents the bone cortex opposite to the site of impact, where the strain gauge is mounted in the experiments. Continuity of cortical bone in the model is through two rods connecting the edges of these beams (which represent lateral bone cortices). The trabecular bone structure is simplified in the model as rods that fill the space between the elastic beams (Fig. 5a). For a "normal" bone condition, as in Fig. 5a, trabecular bone uniformly fills the space between cortices. Accordingly, during impact, and within the elastic small strain domain, the impact force *F*(*t*) can be assumed to be distributed approximately uniformly along the opposite cortex, and the bending moment at the center of the bottom beam where the "strain gauge" is attached, is therefore (Fig. 5a, right frame) [38]:

The corresponding elastic (small) tensile strain at the site of the "strain gauge" in the model is calculated from the theory of beam bending and Hooke's law [38]:



where *h *is the thickness of the elastic beam, *E *is its modulus of elasticity, and *I *is the second moment of inertia around the beam's neutral axis. The strain rate expected at the site of the "strain gauge" in the "normal" bone model, under the small strain and elastic behavior assumptions, is therefore



Now we consider a second case where (osteoporotic) loss of trabecular bone has occurred. For the purpose of modeling, we assume that central trabecular content was lost symmetrically with respect to the point of application of *F*(*t*), so that a void now exists between the two beams that represent cortical bone (Fig. 5b). For a void with length of *L*-2*x *where *x*>0 (Fig. 5b, left frame), the bending moment at the center of the beam is [38]:



For this case, the corresponding elastic tensile strain at the site of the "strain gauge" in the model is [38]:



and the strain rate is



The ratio between  and  when the model is subjected to the same *F*(*t*) is:



However, since 2*x *must be smaller than *L*, the ratio of  over  in Eq. A.7 must be less than unity. Hence, it follows that  must be smaller than , which is, indeed, consistently shown in the experimental data presented in this paper. Moreover, Eq. A.7 shows that the strain rate in the osteoporotic bone  drops proportionally to the size of the void *L*-2*x*, so that when the void is wider (or, when *x *is smaller),  drops more dramatically with respect to . However,  must remain greater than zero, even if all trabecular bone content is missing (Fig. 5c), as long as there is continuity of bone cortices to transfer the bending moment of impact across the specimen.

To summarize, we showed, using a simple analytical biomechanical model, that strain rate at the bone cortex opposite to the site of impact must drop if trabecular bone content is lost, and that the extent of drop is proportional to the amount of lost trabecular bone. On the other hand, continuity of bone cortices requires that the cortical strain rate remains above zero even if all trabeculae were removed. A decreasing sigmoid relation between cortical strain rate and removed mass of trabecular bone, used herein to describe the empirical data, adequately represents this behavior.

## Competing interests

The author(s) declare that they have no competing interests.

## Authors' contributions

TR carried out the impact studies, and drafted the manuscript. AG conceived the study, designed and supervised the experiments, coordinated the study, and prepared the final submitted manuscript. All authors read and approved the final manuscript.
